# Constitutive emission of the aphid alarm pheromone, (*E*)-β-farnesene, from plants does not serve as a direct defense against aphids

**DOI:** 10.1186/1472-6785-10-23

**Published:** 2010-11-23

**Authors:** Grit Kunert, Carolina Reinhold, Jonathan Gershenzon

**Affiliations:** 1 Department of Biochemistry, Max-Planck Institute for Chemical Ecology, Hans-Knöll Str. 8, 07745 Jena, Germany

## Abstract

**Background:**

The sesquiterpene, (*E*)-β-farnesene (EBF), is the principal component of the alarm pheromone of many aphid species. Released when aphids are attacked by enemies, EBF leads aphids to undertake predator avoidance behaviors and to produce more winged offspring that can leave the plant. Many plants also release EBF as a volatile, and so it has been proposed that this compound could act to defend plants against aphid infestation by 1) deterring aphids from settling, 2) reducing aphid performance due to frequent interruption of feeding and 3) inducing the production of more winged offspring. Here we tested the costs and benefits of EBF as a defense against the green peach aphid, *Myzus persicae*, using transgenic *Arabidopsis thaliana *lines engineered to continuously emit EBF.

**Results:**

No metabolic costs of EBF synthesis could be detected in these plants as they showed no differences in growth or seed production from wild-type controls under two fertilizer regimes. Likewise, no evidence was found for the ability of EBF to directly defend the plant against aphids. EBF emission did not significantly repel winged or wingless morphs from settling on plants. Nor did EBF reduce aphid performance, measured as reproduction, or lead to an increase in the proportion of winged offspring.

**Conclusions:**

The lack of any defensive effect of EBF in this study might be due to the fact that natural enemy attack on individual aphids leads to a pulsed emission, but the transgenic lines tested continuously produce EBF to which aphids may become habituated. Thus our results provide no support for the hypothesis that plant emission of the aphid alarm pheromone EBF is a direct defense against aphids. However, there is scattered evidence elsewhere in the literature suggesting that EBF emission might serve as an indirect defense by attracting aphid predators.

## Background

Volatile compounds emitted from plants have been shown to play a variety of roles in protection against herbivores, serving as both direct and indirect agents of defense (e.g. [1-5]). The largest group of plant volatiles is the terpenes, a diverse class of secondary metabolites which includes monoterpenes (C_10_) and sesquiterpenes (C_15_) [6]. One plant-produced sesquiterpene, (*E*)-β-farnesene (EBF), has been considered as a potential defense against aphids since this substance is also the most common constituent of the aphid alarm pheromone [7-12].

Released when aphids are attacked by natural enemies and perceived with the rhinaria of the aphid antenna, EBF leads to predator avoidance behaviors, such as dropping off the plant or walking away [13-15]. Furthermore, EBF is also involved in wing induction in aphids due to natural enemies [16]. While pea aphids (*A. pisum*) are dropping off the plant after EBF perception, or walking away and subsequently searching for a new feeding site, they will encounter other aphids more frequently which leads to wing induction. Having wings gives aphids a good option to leave an area of high predation pressure. The aphid alarm pheromone is also used as a direct defense against natural enemies. By gluing together the mouthparts of predators at least temporarily [17], it allows aphids to escape. In general, the aphid alarm pheromone with its principal component EBF helps aphids to escape from enemy attack in multiple ways.

Given the behaviors elicited by EBF, it can be imagined that plants could employ this sesquiterpene as a defense against aphid infestation [1,18]. Indeed the rate of EBF emission from plants appears to be in the same range as the rate of emission from aphids although the evidence is still very incomplete [19-21]. EBF might act to minimize aphid damage in several ways:

(1) EBF emission may directly prevent aphid settling. This has been reported for the wild potato (*Solanum berthaultii*) which repels the green peach aphid (*Myzus persicae*) by the release of EBF [22,23]. EBF also appears to reduce settling of the pea aphid and blue alfalfa aphid on alfalfa [24]. However, this behavior is strongly affected by (*E*)-β-caryophyllene, another sesquiterpene volatile emitted by many plant species.

(2) EBF might also reduce aphid growth rate by disrupting feeding. Since EBF increases aphid alertness and the time spent walking or dropping off the plant [13,25], EBF-perceiving insects may feed less and so have fewer resources for reproduction, but this possibility has not been investigated.

(3) EBF-induced wing formation might reduce aphid population size. For the pea aphid, it is known that perception of EBF results in a higher percentage of winged offspring than for pea aphids that do not perceive EBF [16]. Since winged offspring leave their host plant before starting reproduction, plants which produce EBF could reduce their aphid load, but this has also not been studied.

Even if a plant trait, such as EBF emission, acts against herbivores, this does not necessarily mean, that it will be beneficial to the plant. Benefits will only accrue when the advantage of the defense exceed its metabolic and ecological costs [5,26].

Metabolic costs arise because the resources required for synthesis, storage and maintenance of plant defenses divert energy that otherwise would be allocated to growth, reproduction or storage [27,28]. Hence to fully evaluate the value of a trait in defense requires an assessment of both costs and benefits.

Here transgenic, EBF-emitting *A. thaliana *and the generalist-feeding green peach aphid (*Myzus persicae*) were used to investigate the potential value to plants of using the aphid alarm pheromone as a direct defense against aphids. After EBF emission from the plants was characterized, the metabolic costs of EBF production in the absence of aphids were quantified. Finally, the potential role of EBF in defending plants against aphids was tested by evaluating its ability to deter settling, reduce performance and induce winged forms.

## Methods

### Plant rearing

Two different lines of transgenic *Arabidopsis thaliana *(L.) Heynh. in a Columbia-0 background were used that constitutively express the *Mentha *x *piperita *(*E*)-β-farnesene synthase gene with the cauliflower mosaic virus 35 S promoter [29]. Plants used in the experiments were progeny of the lines FS11-4 and FS9-2 mentioned in Beale et al. [29] here designated as FS11 and FS9, respectively. Wild-type (WT) ecotype Col-0 plants were used as controls. All plants were grown separately in 10 cm pots in climate chambers (19°C - 21°C, 62% - 70% relative humidity, 110 μmol m^-2 ^s^-1 ^illumination) under short day conditions (10 h light: 14 h dark) for 2-4 weeks (depending on the experiment, see table 1) and afterwards under long day conditions (16 h light: 8 h dark). Plants were grown on soil fertilized with Osmocote® Exact Mini (Scotts International B.V., Heerlen, NL) and Triabon® (COMPO GmbH & Co. KG, Münster, D). Depending on the experiment (table 1), the high fertilizer treatment consisted of 75 g or 100 g/100 l soil for each fertilizer, while the low fertilizer treatment consisted of 12.5 g or 25 g/100 l. The high fertilizer treatment was chosen because initial trials revealed that such a fertilizer concentration promoted maximum growth under the light, temperature and moisture conditions employed. Additional fertilization did not lead to a better growth of the plants. By contrast, the low fertilizer treatment severely reduced growth as judged by plant size. All plants used in the experiments were in a vegetative state (non-flowering) unless otherwise specified.

**Table 1:** Plant growth conditions that varied among experiments

Experiment	Time under short day conditions	Time under long day conditions	Amount of both fertilizers used [g/100 l soil]
1.1. General EBF emission	4 weeks	4 days	all high (100)
1.2. EBF emission under aphid infestation with different fertilizer treatments	3 1/2 - 4 weeks	3 - 5 days	low (12.5)/high (75)
2. Cost of EBF emission	4 weeks	until the end of experiment	low (12.5)/high (75)
3.1. Choice experiment	2 weeks	2 1/2 - 3 weeks	all high (75)
3.2. Aphid performance	4 weeks	3 days	all high (100)
3.3. Wing induction	4 - 4 1/2 weeks	4 - 10 days	all low (25)

### Aphid rearing

The generalist aphid *Myzus persicae *Sulzer was used for all experiments. Insects originated from a culture initiated from individuals collected on tobacco plants in Hannover, Germany. Since 2005 this culture has been reared on *A. thaliana *Col-0 growing under high fertilizer conditions. If not otherwise stated, several lines of aphids were established for each experiment and one line was used for each replicate. To initiate an aphid line, one single adult aphid was placed on one *A. thaliana *WT plant, allowed to reproduce for 2 to 3 days and then removed from the plant. When the offspring reached the adult stage they were transferred to new plants (5 to 6 aphids per plant to avoid crowding) and allowed to reproduce again for 1 to 3 days. This procedure was repeated until enough offspring were available from the line for the experiment. To avoid aphid escape, plants with aphids (and associated control plants without aphids) were covered with cellophane bags (18.8 cm × 39 cm).

### Analysis of volatiles

Volatiles collected as described below on Super Q traps (30 mg Super Q, ARS, Gainesville, FL, USA) were extracted by washing with varying amounts of *n*-hexane (depending on the experiment, see below) containing cuparene as internal standard. To identify compounds, samples were analyzed by GC-MS with a Hewlett-Packard 6890 gas chromatograph coupled to a Hewlett-Packard 5973 quadrupole-type mass selective detector operated in electron impact mode. The mass detector had a transfer line temperature of 230°C, a source temperature of 230°C, a quadrupole temperature of 150°C, an electron energy of 70 eV, and a scan range of 50-400 amu. Helium was used as a carrier gas at a linear flow rate of 2 ml min ^-1^. All samples were separated on a DB-5MS column (J & W, Folsom, CA, USA), of dimensions 30 m × 0.25 mm i.d. × 0.25 μm thick film. The column oven was maintained at 40°C for 2 min then increased at a rate of 5°C min^-1 ^to 160°C followed by 320°C for 2 min. GC retention times of EBF were compared with those of an authentic EBF standard (Bedoukian, Danbury, CT, USA). In addition, mass spectra were compared with those of the National Institute of Standards and Technology Library and the Wiley Library (Hewlett-Packard).

For quantification, 1 μl of each extract was analyzed on a Hewlett-Packard 6890 (Hewlett-Packard, Palo Alto, CA, USA) gas chromatograph equipped with a splitless injector (temperature 220°C) and a flame ionization detector (temperature 250°C). H_2 _was used as a carrier gas at a linear flow rate of 2 ml min^-1^. All samples were analyzed on a DB-5MS column (J & W, Folsom, CA, USA) as specified above. The column oven temperature program was set as described above. (*E*)-β-Farnesene was quantified by comparing the peak area of EBF with that of the internal standard, cuparene (also a sesquiterpene), applying a response factor of 1 [30].

### Experiments

#### 1. Characterization of EBF emission of transgenic *A. thaliana *plants

##### 1.1. Measurement of EBF emission

To characterize the volatile emission of the two different transgenic *A. thaliana *lines (FS11 and FS9), the headspace of two plants (one FS11, one FS9) was simultaneously sampled over 24 h in 2 h intervals. Each plant was placed in a glass chamber of about 27 l where the bottom was closed with guillotine-like Teflon blades. The collection chamber had two holes on the top, one used to inject purified air at a rate of 4 l min^-1^. Eight holes in the lower part of the chamber held the Super Q traps, through which air was pulled out through one trap at a time at a rate of 2 l min^-1^. Volatiles from a 2 h interval were sampled in each trap. Excess air passed through the second hole at the top of the chamber and prevented outside air from entering the chamber. The headspace of 11 plants of each line was sampled and analyzed. Traps were eluted with 200 ng of the internal standard cuparene in 110 μl hexane. After volatile collection, the diameter of each plant was measured.

A direct hexane extraction of the foliage of the transgenic lines was also made to search for stored pools of EBF. However, there were no detectable levels of EBF, suggesting that nearly all of the EBF produced was emitted right after synthesis and not stored in the plant, so that the rate of EBF synthesis is comparable to the rate of emission.

##### 1.2. Influence of aphid infestation and fertilization on EBF emission

Since it is possible that plants reallocate recourses and change their EBF emission depending on fertilization and aphid infestation, the effects of these two factors on emission from both the transgenic lines and wild-type *A. thaliana *were tested. Thus, Arabidopsis WT plants and transgenic FS11 and FS9 plants were grown under high and low fertilizer conditions. Plants from both fertilizer treatments were either infested with aphids or left as uninfested controls. One replicate consisted of 12 plants (3 plant genotypes × 2 fertilizer treatments × 2 aphid treatments). Five replicates were investigated. Aphids for one replicate did not originate from a single aphid line as described above but from 10 adult aphids distributed on two plants. Further aphid rearing was according to the method described above. Three days before the start of the volatile collection, 20 aphids of the 4^th ^larval stage were placed on the plants. During these three days they became adults and started to produce offspring. For the 24 h volatile collection, plants were separately placed in 3 l glass desiccators. The desiccators were closed with glass lids containing three openings. One was used to pump in purified air at a rate of 3 l min^-1^, another opening contained the Super Q filter through which air was pulled out at a rate of 2 l min^-1^, and excess air passed through the third opening to prevent contamination from outside air.

Super Q filters were eluted with 1 μg cuparene (internal standard) in 160 μl hexane. After the volatile collection, plant diameter was measured and all aphids were removed and frozen for later counting.

#### 2. Cost of EBF emission

In order to test whether EBF emission is costly to the plants, emission, plant growth, and seed production were measured over the lifetime for the 3 different plant genotypes growing under 2 different fertilizer regimes. Each replicate consisted of 6 plants (3 genotypes × 2 fertilizer regimes). In total 40 replicates were investigated. Measurement of plant diameter started 2 weeks after sowing and was repeated every week until the death of the plant. Volatiles were collected for 24 h from a randomly-chosen subset of 6 replicates, starting with 3 week-old plants and this was repeated every second week with the same plants through flowering until plants had completed their life cycle. For the volatile collection, plants were transferred to 3 l glass desiccators as described in the previous experiment. However, in this experiment the desiccators were tightly closed. Purified air entered the desiccator at a rate of 2 l min^-1^, came into contact with the plant and left the vessel through the Super Q trap. Traps were eluted with an internal standard of 1 μg cuparene in 150 μl hexane. EBF emission for the whole lifetime of a plant was estimated by the following equation:

$$\text{lifetime EBF}=14*\Sigma {\text{ EBF}}_{\text{day x}}$$

with EBF_day x _is the amount of EBF measured every second week for 24 hours. 14 represents the sampling interval in days.

To determine seed production, stems with siliques were wrapped in paper bags fixed on a wooden stick. Plants were watered and allowed to grow until the rosette was dead. Plants were then kept dry for about 3 weeks. During this time siliques opened and released the seeds in the bag. Seeds were later separated from other plant material and weighed.

#### 3. Benefits of EBF emission

##### 3.1. Choice experiment

In order to test whether plants benefit from EBF emission due to avoidance of EBF emitting plants by aphids, the 3 different genotypes (WT, FS11, FS9) were tested in two choice experiments, one with winged and one with wingless aphids. Since aphids are known to orientate visually, it was first tested whether plants differ in leaf reflectance. The spectral reflectance of the upper side of source leaves was measured with a Perkin Elmer Lambda 950 spectrometer. Measurements started at a wavelength of 350 nm and were continued until 700 nm in 5 nm steps. Five plants of each genotype were used and three randomly chosen source leaves per plant were measured. The mean values of the three leaves per plant were calculated and used for the analysis.

The aphids' preference towards the plant (70 cm × 70 cm × 70 cm) equipped with a lid containing 6 circular openings (diameter 13 cm) covered with gauze and with 6 holes in the non-transparent bottom where 2 WT, 2 FS11, and 2 FS9 plants were positioned in a way that the leaves were above the surface. Two plants of the same genotype were placed opposite to each other. To verify that EBF distributed in the arena headspace was correlated with the positions of plants emitting EBF, a solid phase micro extraction (SPME) fiber (100 μm Polydimethylsiloxane coating) was placed above one WT, one FS 11 and one FS 9 plant in the arena for 6.5 hours. Afterwards the SPME fibers were manually desorbed in the inlet of the GC-MS, and volatiles were analyzed as described above. In order to look specifically for EBF the mass spectrometer was run in the selected ion monitoring mode (m/z 69 + 93 + 133) instead of the scan mode as was done for the other volatile analyses. EBF was detectable above plants of the high-emitting FS 9 plants in higher amounts than above plants of the low-emitting FS 11 plant. No EBF at all was detected near the WT plant (additional file 1 and 2).

After spectral characterization of the plant surface, aphids were tested in an arena for their preference towards the plant genotypes. Wingless aphids were reared as described above. To obtain winged individuals, a crowding treatment was imposed in which 15 aphids of a line were put on one plant and allowed to reproduce for 2 weeks. Afterwards all aphids (at this time still wingless) were transferred to a new plant where they were allowed to reproduce for another week. After this time, enough winged individuals for one replicate were available.

Both choice experiments were performed in the above mentioned Plexiglas arena. For each of the 20 replicates, new plants were used and the position of the plants was changed. The distance between the plants was 25 cm, which was also the distance between the middle of the arena (aphid release point) and the middle of the plants. For each replicate, 15 adult aphids were put in a Petri dish (5.5 cm diameter). The lid of the Petri dish was fixed to a stick which allowed removing the lid without opening the lid of the arena. For wingless aphids, the Petri dish was put in the middle of the arena on the bottom, while for winged aphids the Petri dish was placed on the top of a pole of 35.5 cm height. After opening the lid of the Petri dish, aphids had 24 h to choose a plant. After this time, the plants were taken out of the arena, scanned for aphids and their diameters were measured. To avoid optically-biased orientation of the aphids, all sides of the arena were covered with white paper.

##### 3.2. Aphid performance experiment

To investigate whether aphids living on EBF emitting plants might have a lower reproductive success due to a higher restlessness and therefore less feeding time, offspring production was tested on all three plant genotypes. For one replicate, 15 adult aphids of one line were placed on WT, FS11 and FS9 plants, respectively, and allowed to reproduce for 24 h. After this time the adult aphids were transferred to a new set of plants and again allowed to reproduce for 24 h. This was repeated another two times. After the third transfer (fourth set of plants) the adult aphids were removed. Plants with offspring were kept until the offspring became adults. These adults were then removed from the plants and frozen for later counting and morph determination. The experiment consisted of 20 replicates.

##### 3.3. Aphid wing induction experiment

In order to examine whether the green peach aphid is able to produce more winged offspring when perceiving EBF, 80 aphids per replicate of the 4^th ^larval stage or young adult stage were transferred to two Arabidopsis WT plants (40 aphids per plant). One plant served as a treatment plant and for three days aphids on this plant were treated with a 3 μL solution twice a day containing 100 ng EBF dissolved in *n*-hexane (first phase of experiment). The other plant was used as control plant and aphids on this plant were treated in the same way as on the treatment plant except that the applied solution contained only 3 μl *n*-hexane. The solution was applied through a small hole in the cellophane bag on a piece of filter paper which was fixed to a toothpick. After three days, adult aphids were transferred to a new plant and again treated with EBF solution as before (second phase of experiment). After another three days, adult aphids were removed and offspring on the plants were kept until they became adult. Then they were removed and frozen for later counting and morph determination. In total, 16 replicates were investigated.

### Statistical analyses

Results are presented as mean ± SE. To investigate the influence of plant genotype, fertilizer and/or aphid presence on plant size, seed production, EBF emission, and leaf reflectance, analyses of variance were used. Data for leaf reflectance were arcsine square-root transformed to improve the normality of the residuals. Data of EBF emission over the lifetime of the plants were log transformed for normalization. The influence of plant genotype and plant size (diameter), and the influence of plant genotype, fertilizer, and number of aphids on EBF emission were tested with analyses of covariance with diameter and number of aphids as continuous variables. For these tests only the EBF emitting plants were used.

Generalized linear models (glm) were applied to find out the influence of plant genotype, fertilizer and plant diameter on the number of aphids, and to find out whether EBF application influenced aphid reproduction in the wing induction experiment. In order to deal with overdispersion, a quasipoisson distribution was used in the models. The influence of the EBF application treatment and aphid number on wing production was also tested with a generalized linear model, but with a quasibinomial error family to account for the binomial error structure and overdispersion.

To test, whether aphids prefer WT plants over EBF emitting plants, a mixed effect model with plant genotype and plant diameter as fixed effects, and the position of the plant inside the arena and the replicate as random effects was applied. Percentage data were arcsine square-root transformed for the improvement of the residuals' normality. A similar model was used to test whether plant size differs between the plant genotypes in this experiment. A mixed effect model with temporal pseudoreplication was used with plant genotype as fixed effect and the day of measurement, which represents a pseudoreplication within each aphid line, as a random effect. Whenever possible, models were simplified by removing non-significant terms [31]. All analyses were done in R 2.10.0 [32].

## Results

### 1. Characterization of EBF emission of transgenic *A. thaliana *plants

#### 1.1. Measurement of EBF emission

**Figure 1:**
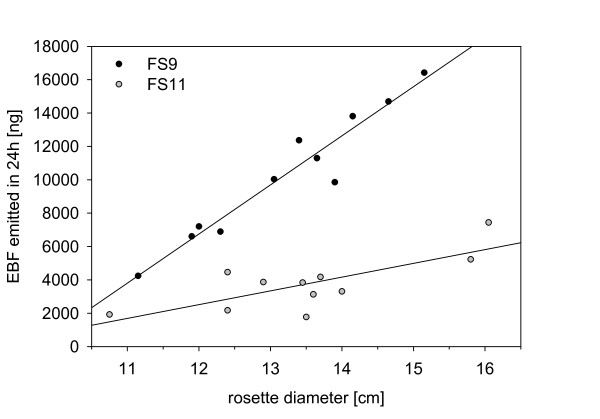
**The relationship between the EBF emission of a plant over 24 h and its rosette diameter**. Regression line FS11: amount of EBF released [ng] = -7387.1 + 825.0 × rosette diameter [cm], (P = 0.007, r^2 ^= 0.569, n = 11); Regression line FS9: amount of EBF released [ng] = -28581.1 + 2944.3 × rosette diameter [cm], (P < 0.001, r^2 ^= 0.931, n = 11)

The two transgenic lines (FS11, FS9) both produced EBF during the entire diurnal cycle (additional file 1 and 3), with FS9 plants emitting more EBF than FS11 plants (F = 196.89, p < 0.001). Approximately 200 ng (FS11) and 700 ng EBF h^-1 ^per plant (FS9) were released at the peak emission time at the end of the light period. These rates are comparable to those previously reported for plants from these same lines [25]. While for both genotypes plants of a greater size (larger rosette diameter) emitted more EBF (F = 91.55, p < 0.001), large FS9 plants released proportionally more EBF than large FS11 plants (interaction between plant genotype and diameter: F = 34.56, p < 0.001, Figure 1). No EBF was detected from wild-type plants in this experiment which was conducted only on vegetative stage plants.

#### 1.2. Influence of aphid infestation and fertilization on EBF emission

Since the experimental treatments to study cost and benefits of EBF emission involved differential fertilization and aphid infestation, the effects of these two factors on emission from both the transgenic lines and wild-type *A. thaliana *were tested. Fertilization was not found to significantly influence the *mean number of aphids *(F = 0.69, p = 0.413) although there was some tendency to have more aphids on highly fertilized plants with the means ranging from 134.8 ± 14.7 on low fertilizer WT plants to 156.8 ± 17.3 on high fertilizer FS9 plants. In addition, the aphid number on the plant genotypes did not differ (plant genotype: F = 0.05, p = 0.946) and was not dependent on the size of the plant (diameter: F = 0.09, p = 0.770).

*The rosette diameter *of the plants changed with fertilizer treatment with plants growing larger when better fertilized (11.8 cm ± 0.2 cm vs. 7.8 cm ± 0.2 cm, F = 239.2, p < 0.001) but diameter was neither different between plant genotypes (F = 0.43, p = 0.654) nor influenced by aphid infestation (F = 0.02, p = 0.886).

**Figure 2:**
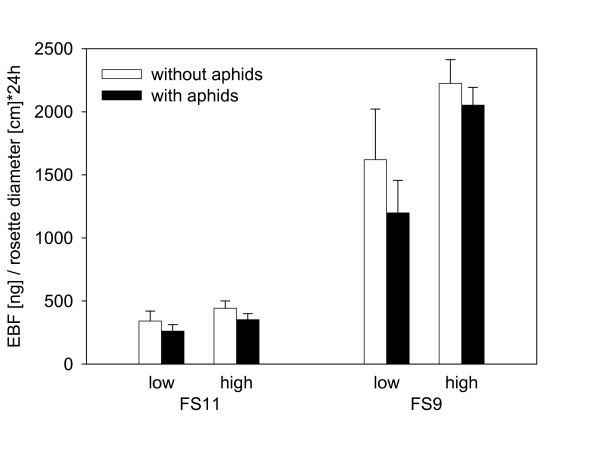
**The amount of EBF emitted during 24 h for plants growing under high and low fertilizer conditions with and without aphid infestation**. The bars show mean values ± SE of 5 replicates.

*EBF emission *could not be detected in the headspace of *A. thaliana *WT plants regardless if they were aphid infested or not. Fertilizer treatment influenced EBF emission with high fertilizer plants emitting significantly more EBF than plants which were grown under low fertilizer conditions (F = 51.21, p < 0.001). This increase ranged from 30 - 70% based on rosette diameter. However, the number of aphids on a plant did not have a significant influence on its EBF emission (F = 2.24, p = 0.143, Figure 2). As in the first experiments, EBF emission was again dependent on plant genotype and size with FS9 plants emitting more EBF then FS11 plants (F = 191.10, p < 0.001) and larger plants emitting more EBF than smaller plants (F = 13.38, p < 0.001).

### 2. Cost of EBF emission

**Figure 3:**
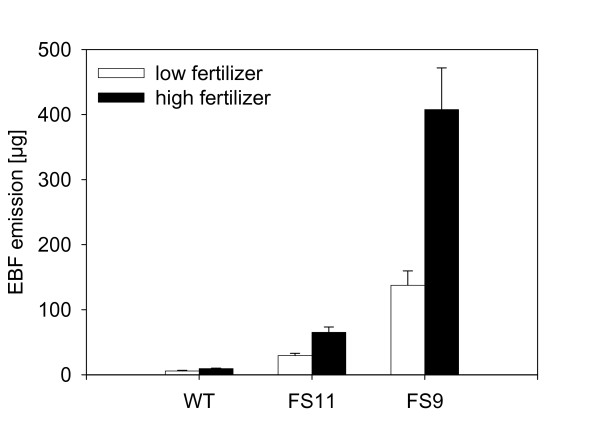
**Estimated EBF emission over the lifetime of *A. thaliana *plants growing under high and low fertilizer conditions**. Bars show mean values ± SE of 6 replicates.

**Figure 4:**
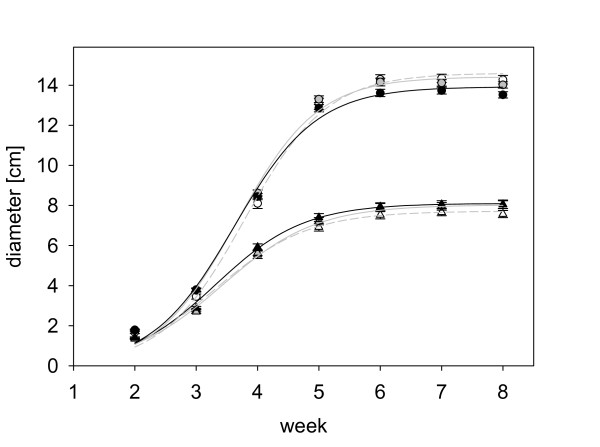
**The growth of EBF emitting and wild type plants over time under different fertilizer regimes**. Triangles represent low fertilizer conditions, circles high fertilizer conditions. Empty symbols stand for wild type plants (Col-0), grey symbols for FS11 plants, and black symbols for FS9 plants. Lines represent the logistic growth functions for the different treatments (diameter = a/(1 + exp (b - c * week))). Parameters: low fertilizer conditions: wild type plants: a = 7.72, b = 4.39, c = 1.32; FS11 plants: a = 8.03, b = 4.47, c = 1.31; FS9 plants: a = 8.10, b = 4.61, c = 1.39; high fertilizer conditions: wild type plants: a = 14.61, b = 5.67, c = 1.50; FS11 plants: a = 14.42, b = 5.57, c = 1.52; FS9 plants: a = 13.92, b = 5.39, c = 1.49.

**Figure 5:**
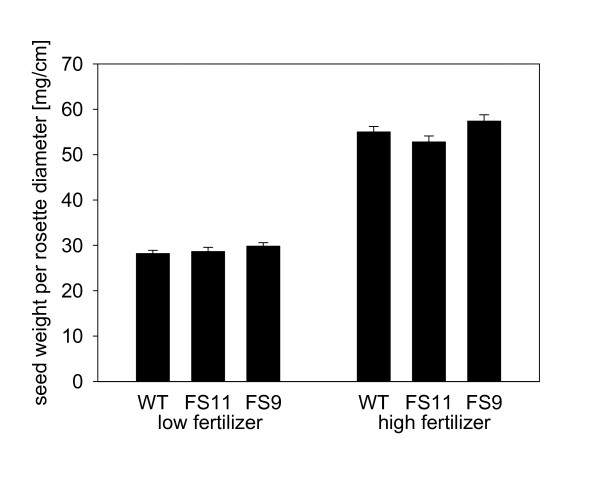
**Seed mass produced by EBF emitting and wild type plants grown under high and low fertilizer conditions**. Bars present mean values ± SE.

In order to test whether EBF emission is costly to plants in the absence of aphids, emission, growth and seed production were measured over the lifetime of the two transgenic, EBF-emitting lines and wild-type *A. thaliana *grown under two different fertilizer regimes. The estimated amount of EBF emitted during the lifetime of the plants differed significantly between genotypes (F = 244.20, p < 0.001) with FS11 plants emitting less than FS9 plants and wild-type plants emitting only very small amounts of EBF during their flowering stage. High fertilizer treatment plants emitted much more EBF than plants growing under low fertilizer conditions (F = 41.64, p < 0.001, Figure 3). However, there was no evidence of a cost of emission in these transgenic lines. The final sizes of the different plant genotypes, FS9, FS11 and wild-type, did not differ (F = 0.96, p = 0.385, Figure 4), and the seed weight produced per rosette also showed no difference among genotypes (F = 2.25, p = 0.108, Figure 5). Differences in the final plant size and the seed weight could only be detected between the two fertilizer treatments (final size: F = 1675.25, p < 0.001, Figure 4; seed weight: F = 921.32, p < 0.001, Figure 5) where high fertilizer plants became approximately twice as big as plants growing under low fertilizer conditions and also had a seed weight nearly twice as high.

### 3. Benefit of EBF emission

#### 3.1. Choice experiment

**Figure 6:**
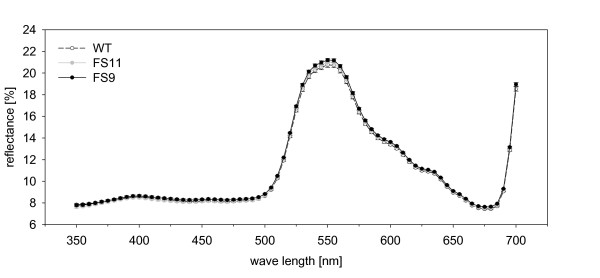
**Leaf reflectance of the upper side of source leaves of wild type and EBF emitting plants**.

Since aphid host choice may be influenced by the wavelength of reflected light, the three studied genotypes were compared on the basis of their reflectance over the spectrum from 350-700 nm. However, the genotypes did not differ in leaf reflection at any wavelength (all p values > 0.05, Figure 6).

**Figure 7:**
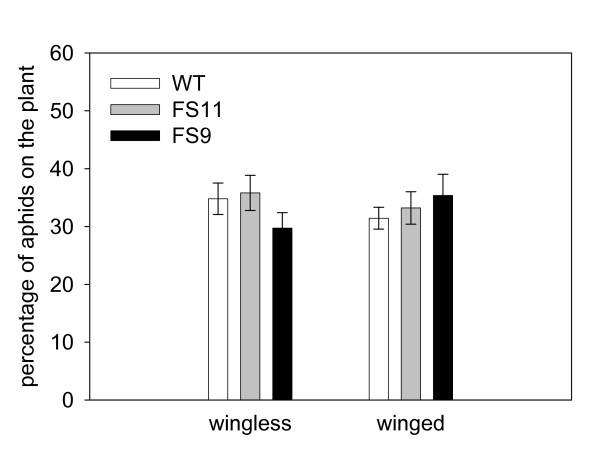
**Preference of aphids for EBF-emitting or wild-type plants in the choice experiment**. Bars present mean values ± SE.

In the choice experiment, neither aphid morph (winged or wingless aphids) differentiated between the plant genotypes (wingless aphids: F = 0.49, p = 0.611; winged aphids: F = 0.30, p = 0.739; Figure 7). Plant diameter did not differ significantly between genotypes (experiment with wingless aphids: F = 1.01; p = 0.367; experiment with winged aphids: F = 1.77, p = 0.176) and diameter did not influence aphid choice (wingless aphids: F = 0.78, p = 0.380; winged aphids: F = 0.06, p = 0.816).

#### 3.2. Aphid performance experiment

**Figure 8:**
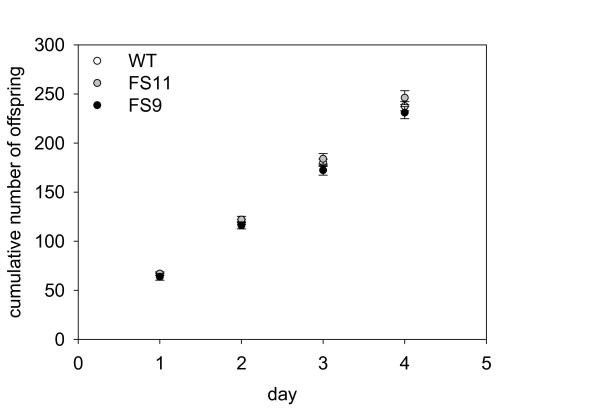
**Cumulative number of offspring of 15 adult aphids reared on wild type and EBF emitting plants**.

Plants may also derive benefit from EBF emission if aphid perception of the alarm pheromone reduces their feeding and decreases reproduction. However, the number of aphid offspring produced on EBF-emitting and wild-type control plant lines did not differ significantly (F = 0.31, p = 0.733, Figure 8). Winged offspring occurred only sporadically (5 winged individuals in the whole experiment).

#### 3.3. Aphid wing induction experiment

**Figure 9:**
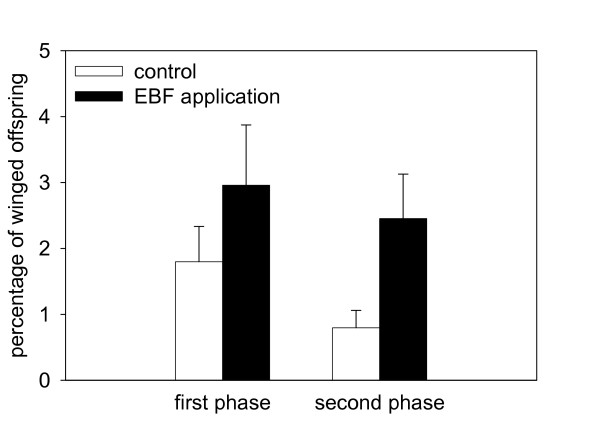
**Percentage of winged morphs among offspring born by mothers exposed to 100 ng of EBF twice a day for three days**. The first phase represents the first 3-day interval, the second phase the second 3-day interval of the experiment, where aphids were treated with EBF or the control solution. The bars show the mean values ± SE.

To verify whether *M. persicae *could produce more winged offspring after perceiving EBF, as shown previously for the pea aphid [16], pure EBF was used to treat aphids on *A. thaliana *wild-type plants. After EBF application typical predator avoidance behavior like kicking and running away could be observed. The proportion of winged offspring among offspring born during the first 3 days of the experiment (first phase of experiment) was slightly higher in the EBF treatment than in the control treatment, but this difference was not significant (F = 1.27, p = 0.268, Figure 9). However, during the second 3 days of the experiment (second phase) aphids treated with EBF produced significantly more winged offspring than aphids in the control treatment (F = 8.28, p = 0.007, Figure 9). The number of offspring did not significantly influence the wing induction in the first phase (F = 0.57, p = 0.461) or in the second phase (F = 2.00. p = 0.168). Moreover, the number of offspring did not differ significantly between the treatments (first phase: mean EBF treatment 357.2 ± 23.8, mean control treatment 388.7 ± 21.0, F = 0.98, p = 0.330; second phase: mean EBF treatment 416.6 ± 16.5, mean control treatment 444.5 ± 21.6, F = 1.06, p = 0.312).

## Discussion

Emission of the aphid alarm pheromone could serve as a defense against aphids, but its value to plants depends on both the costs and benefits of emission. In previous work with the transgenic *A. thaliana *lines studied here, Beale et al. [29] could detect no metabolic costs based on the similar size, growth rate and flowering time of EBF-emitting lines and wild-type controls. Here it was shown that the growth (Figure 4) of EBF-emitting and wild-type plants were also not different from each other under two fertilizer regimes where the low fertilizer regime caused a sharp reduction in plant growth. In addition, we demonstrated that seed production also did not differ among lines emitting EBF and wild-type controls (Figure 5). Thus, there is no evidence for any metabolic costs associated with EBF production in these lines. This conclusion may apply to other plant species as well since the rate of EBF emission from the *A. thaliana *transgenic lines is in the same range as that of EBF emission reported for other plant species [19-21]. The lack of metabolic costs may simply be due to the low rate of production. The amount emitted in 24 hours corresponds to less than 0.1 ‰ of the fresh weight of the above ground biomass. However, metabolic costs of EBF emission might conceivably be observed under other stress conditions, such as greater nutrient limitation, low light, drought or various biotic stresses [33-35]. Volatile emission can also attract additional herbivores and can therefore generate ecological costs [36]. The biosynthesis of EBF and other sesquiterpenes requires substantial amounts of fixed carbon and energy for substrates and cofactors [37]. Additionally, the diversion of farnesyl diphosphate (FPP) to EBF synthesis may directly reduce the supply of sterols (a major group of membrane components) and other isoprenoid metabolites produced from FPP. Beale et al. [29] noted that, when flowering, EBF-producing *A. thaliana *plants emit lower amounts of other sesquiterpenes than wild-type plants.

The transgenic, EBF-producing *A. thaliana *lines expressed the EBF synthase gene under the control of a constitutive promoter. Hence the enzyme should be present in almost every cell and produce EBF whenever and wherever FPP is available. From this perspective, the level of EBF produced is an indicator of the size of the FPP pools. Given the increase in emission during the day as compared to the night period (additional file 3), these pools appear to be larger during the light phase, possibly due to the action of photosynthesis. Of the two basic isoprenoid pathways operating in plant cells, the MEP pathway is strongly stimulated by light [38]. A direct relationship between the rate of photosynthesis and the rate of EBF formation is consistent with the trend observed for greater emission from larger plants (Figure 1), which presumably have more active photosynthetic leaf area. Fertilization also promoted EBF formation (Figure 3), probably by increasing plant size (Figure 4).

Since no metabolic costs of EBF emission were detectable, any effect of EBF against aphids should be advantageous for the plant fitness as long as there are no high ecological costs involved. It was tested whether EBF emission could protect plants from aphids in several ways, by (1) repelling insects, (2) reducing their reproduction and (3) inducing wing formation. In tests for repulsion, both winged and wingless aphids were used since it is known, that winged morphs (migrants) can respond differently from wingless morphs to visual and olfactory stimuli [1] due to differences in their sensory systems [39]. However, neither morph was repelled by constant EBF emission from the transgenic lines, and neither morph distinguished between emitting and control plant lines (Figure 7) under the conditions employed. One reason for this behavior could be that aphids chose the plant at night when EBF emission is strongly reduced. However, aphids are known to be active during the day time [40], and were observed to start searching for a host plant shortly after the beginning of the experiment in the light period.

In addition to olfaction, visual cues are important in aphid host choice, especially for winged aphids [41] and landing involves a phototactic response to the wavelengths reflected by the plant [42]. For *M. persicae*, three spectral types of photoreceptors are known (near UV: 330 - 340 nm; blue - green: 490 nm; and green: 530 nm) [43] and yellow colors act as very strong stimuli [42,44]. If the plants tested had differed in their leaf reflectance, this cue could have influenced the aphid's host plant choice. However, leaf reflectance of EBF emitting plants did not differ from that of wild type plants (Figure 6). The size of the color stimulus is also important in aphid attraction [42], but again EBF emitting plants did not differ in size from wild type plants. Thus, visual cues are not likely to have influenced the outcome of our experiments.

That *Myzus persicae *was not repelled by EBF emission is contrary to previous findings where EBF released from wild potato (*Solanum berthaultii*) was shown to deter aphids [22,23]. This discrepancy might be ascribed to differences between the species in their mode of EBF release. *S. berthaultii *contains two types of glandular trichomes (A and B), with type A trichomes having a four-lobed head which contains EBF and other volatile compounds, while type B trichomes bear a sticky droplet on their tops [23,45,46]. When aphids contact the leaf surface, their tarsi get coated by the sticky exudate of the type B trichomes. While extricating themselves, they destroy the head of type A trichomes [46] which then release the EBF. In this system, EBF is released in pulses whenever a trichome is ruptured, mimicking the EBF emission by aphids when they get attacked by natural enemies. In contrast, the transgenic *A. thaliana *lines tested here appear to release EBF continuously over the diurnal period based on our volatile collection data. The difference between continuous vs. pulsed EBF release might be crucial for aphid deterrence.

Once on a plant, aphids may be disturbed by the release of alarm pheromone and interrupt feeding more often than aphids on wild type plants would do [47,48]. As a consequence aphids on EBF-emitting plants would likely grow and reproduce less. However, in the present study aphid performance on EBF-emitting plants was the same as on wild type plants (Figure 8). This is not due to aphid feeding causing a reduction in EBF release. The emission of EBF was not significantly affected by aphid infestation (Figure 2). The undiminished performance of aphids on EBF-releasing plants is contrary to the findings of Gut et al. [49] who noticed a reduced aphid growth when *Myzus persicae *feeding on cabbage plants covered with a plastic bag were exposed to 10 mg of EBF applied on a filter paper placed next to the plant. But, 10 mg EBF is a very high dose, much more than our transgenic *A. thaliana *plants emitted during their whole lifetime (Figure 3), and 200,000 fold more than an aphid would emit when attacked by an enemy [50,51]. Such a high dose of EBF may have been toxic to the aphids, and therefore reduced their growth.

EBF emission might also benefit plants by leading to the production of more winged offspring which would leave the plant. EBF-caused wing induction has been demonstrated previously for the pea aphid (*Acyrthosiphon pisum*) and arises naturally as a consequence of EBF release by aphids during an enemy attack. The ability of enemy attacks to trigger wing induction has been observed frequently for the pea aphid [52-55] as well as the cotton aphid (*Aphis gossypii*) [56]. The phenomenon is thought to arise as a result of the higher aphid activity on the plant caused by EBF which leads to more frequent encounter rates, similar to what happens when aphid density is high [16]. However, wing formation in this study was not significantly induced in the green peach aphid by EBF emitted from the transgenic *A. thaliana *plants, even though this aphid species is in fact capable of producing more winged offspring when it perceives EBF (Figure 9), which is not universally true for all aphid species [52,57] or clones [58]. The fact that the green peach aphid did produce more winged offspring when treated with two EBF pulses a day for three days compared to aphids which were treated just with the control solvent (Figure 9) was not due to different aphid densities, which have been previously shown to influence wing induction [59,60]. This and the observed predator avoidance behavior of the aphids when EBF was applied demonstrate that the aphids in the experimental colony were still sensitive to EBF. Thus the green peach aphid was able to produce more winged offspring when it perceived pulses of synthetic EBF, but it did not produce more winged offspring if the EBF was being emitted from plants at a constant rate. Taking all the results together, it seems that EBF emitted from transgenic *A. thaliana *was not effective in causing changes in the physiology and behavior of the green peach aphid that have been shown to be triggered by EBF in other studies. This discrepancy may be due to the fact these transgenic plants release EBF constantly as opposed to the pulsed release caused by natural enemy attacks on individual aphids. There are two non-exclusive possibilities for why aphids do not react to continuous emission of EBF. First they might get habituated to the compound after extended exposure which is also known for other insects responding to pheromones [61,62]. In fact, Wohlers [15] determined that pea aphids could become habituated to their alarm pheromone since they did not show typical escape behavior after some time of EBF exposure. Petrescu et al. [63] reached the same conclusion but only applied EBF once in 24 h, and it is unclear whether EBF remained in the vicinity of the aphids long enough so that they could become habituated. The other possibility is that aphids react to EBF only if it is emitted in pulses, which would mimic the release caused by attack on individual members of an aphid colony. This could explain why the green peach aphid reacted to *S. berthaultii *where the EBF was only released as individual EBF-containing trichomes were destroyed [23]. The mode of EBF release, whether pulsed or continuous, might therefore be an important cue in informing aphids whether the EBF is coming from attacked conspecifics (so it is necessary to take evasive action) or from a plant (so there is no immediate predation risk).

## Conclusions

The results of this investigation demonstrate that EBF produced continuously by transgenic *A. thaliana *does not act as a direct defense against aphids. The same conclusion might well be applicable to the continuous emission of EBF from other plants though more studies are necessary. However, EBF might still act indirectly against aphids via the attraction of natural enemies. Some aphid natural enemies have been reported to perceive EBF and be attracted by it [4,29,64-66]. Further long-term studies are needed with EBF-emitting plants to determine if these enemies can effectively reduce aphid load on EBF-emitting plants or whether they get habituated or confused by constitutive EBF emission.

## Authors' contributions

CR carried out the choice experiment and the aphid wing induction experiment. GK planned and conducted all the other experiments and performed the statistical analyses. The whole study was designed and coordinated by GK. GK and JG drafted the manuscript. All authors read and approved the final version of the manuscript.

## Supplementary Material

Additional file 1 **Appendix**. Time course of daily EBF emission, results and figure legends Click here for file

Additional file 2 **Appendix Figure A1**. EBF dispersion in the choice test arena after 6.5 hours. Click here for file

Additional file 3 **Appendix Figure A2**. EBF emission from transgenic *A. thaliana *lines over the course of a single day. Click here for file
