# Supplementary material for: Constitutive emission of the aphid alarm pheromone, (E)-β-farnesene, from plants does not serve as a direct defense against aphids
Source: BMC Ecol. 2010 Nov 23;10:23. doi: 10.1186/1472-6785-10-23 (PMC3002888; doi:10.1186/1472-6785-10-23)
Supplement: Additional file 1 — Appendix. Time course of daily EBF emission, results and figure legends [file 1472-6785-10-23-S1.PDF]

Appendix

Time course of daily EBF emission

Material and Methods

see main text

Results

The two transgenic lines (FS11, FS9) both produced EBF during the entire diurnal cycle with the rate of emission increasing during the day until the light was switched off (10 pm). In darkness, the rate of EBF emission decreased and reached its lowest point at the end of the night period (6 am). This pattern was the same for both genotypes (additional file 3), yet more pronounced in the FS9 genotype.

Figures

Figure A1 - EBF dispersion in the choice test arena after 6.5 hours.

GC-MS analysis of EBF collected from SPME fibres used to sample the headspace above non-EBF emitting WT plants, and above transgenic EBF emitting plants (FS11 and FS 9) in the choice test arena.

Figure A2 – EBF emission from transgenic *A. thaliana* lines over the course of a single day.

Dots represent the mean EBF emission per plant of 11 plants. The shaded area symbolizes night time. Collections were made over 2 hour intervals.
