# Supplementary figures and images for: Constitutive emission of the aphid alarm pheromone, (E)-β-farnesene, from plants does not serve as a direct defense against aphids
Source: BMC Ecol. 2010 Nov 23;10:23. doi: 10.1186/1472-6785-10-23 (PMC3002888; doi:10.1186/1472-6785-10-23)

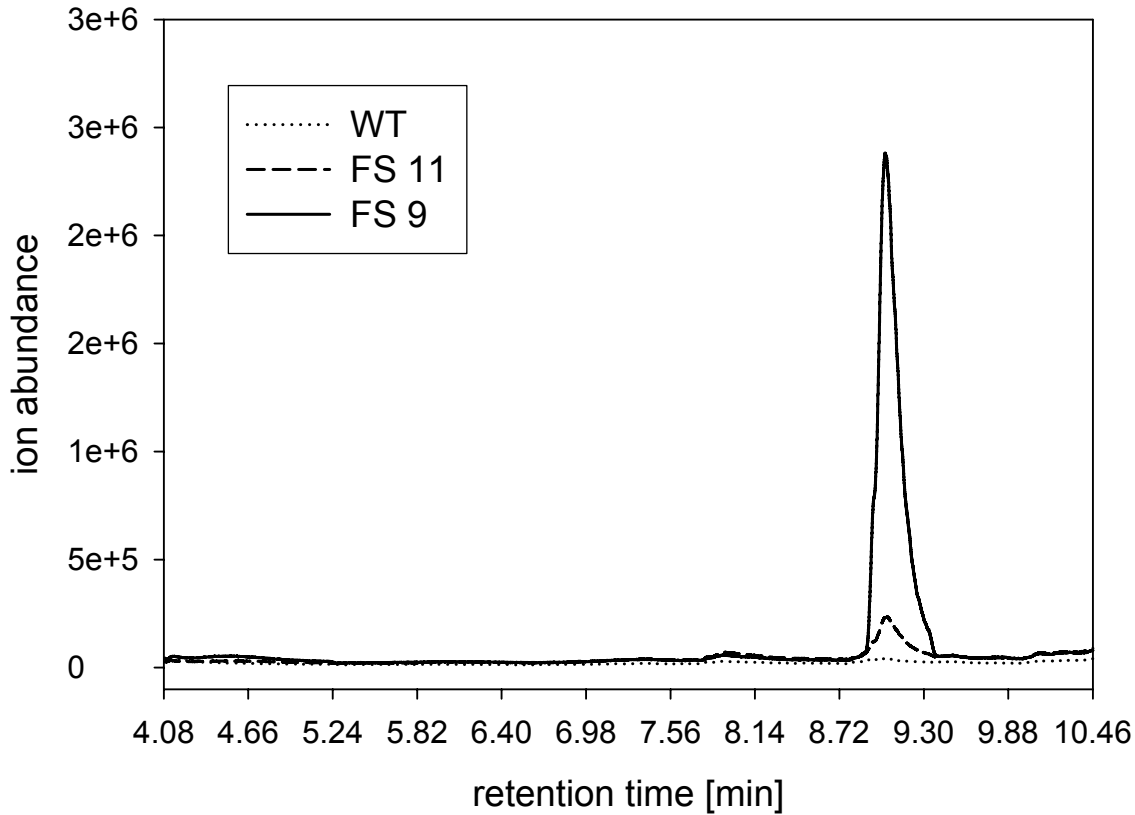

Supplement: Additional file 2 — Appendix Figure A1. EBF dispersion in the choice test arena after 6.5 hours. [file 1472-6785-10-23-S2.PDF]

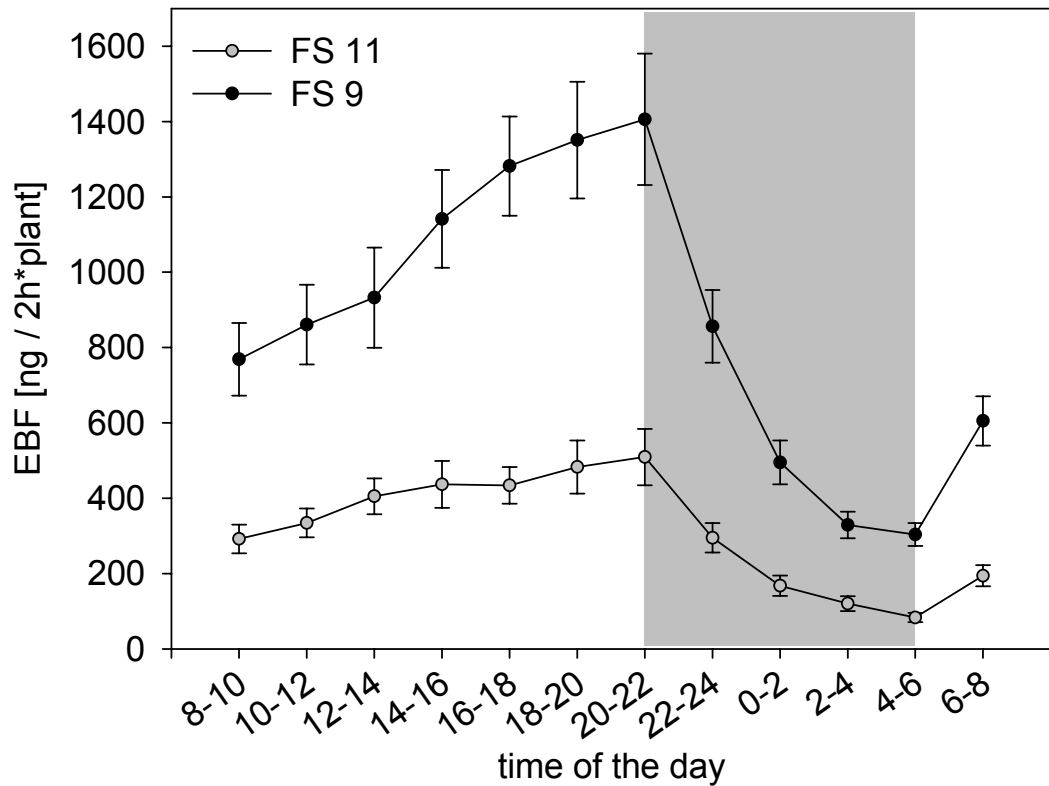

Supplement: Additional file 3 — Appendix Figure A2. EBF emission from transgenic A. thaliana lines over the course of a single day. [file 1472-6785-10-23-S3.PDF]
